# GARP dysfunction results in COPI displacement, depletion of Golgi v-SNAREs and calcium homeostasis proteins

**DOI:** 10.3389/fcell.2022.1066504

**Published:** 2022-12-12

**Authors:** Amrita Khakurel, Tetyana Kudlyk, Irina Pokrovskaya, Zinia D’Souza, Vladimir V. Lupashin

**Affiliations:** Department of Physiology and Cell Biology, University of Arkansas for Medical Sciences, Little Rock, AR, United States

**Keywords:** GARP complex, golgi trafficking, Cab45, COPI, golgi SNAREs, GBF1, proteomics, GOLPH3

## Abstract

Golgi-associated retrograde protein (GARP) is an evolutionary conserved heterotetrameric protein complex that tethers endosome-derived vesicles and is vital for Golgi glycosylation. Microscopy and proteomic approaches were employed to investigate defects in Golgi physiology in RPE1 cells depleted for the GARP complex. Both *cis* and *trans*-Golgi compartments were significantly enlarged in GARP-knock-out (KO) cells. Proteomic analysis of Golgi-enriched membranes revealed significant depletion of a subset of Golgi residents, including Ca^2+^ binding proteins, enzymes, and SNAREs. Validation of proteomics studies revealed that SDF4 and ATP2C1, related to Golgi calcium homeostasis, as well as intra-Golgi v-SNAREs GOSR1 and BET1L, were significantly depleted in GARP-KO cells. Finding that GARP-KO is more deleterious to Golgi physiology than deletion of GARP-sensitive v-SNAREs, prompted a detailed investigation of COPI trafficking machinery. We discovered that in GARP-KO cells COPI is significantly displaced from the Golgi and partially relocalized to the ER-Golgi intermediate compartment (ERGIC). Moreover, COPI accessory proteins GOLPH3, ARFGAP1, GBF1, and BIG1 are also relocated to off-Golgi compartments. We propose that the dysregulation of COPI machinery, along with the depletion of Golgi v-SNAREs and alteration of Golgi Ca^2+^ homeostasis, are the major driving factors for the depletion of Golgi resident proteins, structural alterations, and glycosylation defects in GARP deficient cells.

## Introduction

The Golgi complex is a central hub in the secretory and endocytic pathway (Le Borgne and Hoflack, 1998; Park et al., 2021). It functions in trafficking, processing, and proper sorting of newly synthesized proteins in an anterograde manner while recycling Golgi-resident proteins in a retrograde manner (Johannes and Popoff, 2008; Blackburn et al., 2019). It houses multiple resident proteins, cargo receptors, sugar transporters, and glycosylation enzymes (Stanley, 2011; D'Souza et al., 2020). The Golgi-associated retrograde protein (GARP) complex is an evolutionarily conserved multisubunit protein complex of four different subunits, VPS51, VPS52, VPS53, and VPS54 (Conibear and Stevens, 2000; Oka and Krieger, 2005). It is localized in the *trans*-Golgi network (TGN) and is known to function in tethering vesicles arriving from the late endosomes to the TGN (Pérez-Victoria et al., 2008; Pérez-Victoria and Bonifacino, 2009). GARP shares its three subunits, VPS51, VPS52, and VPS53, with the Endosome-Associated Recycling Protein (EARP) complex (Schindler et al., 2015). The GARP complex is recruited to the TGN by ARL5 GTPase (Ishida and Bonifacino, 2019).

Mutations in GARP complex subunits have been found to cause neurodevelopmental disorders. In humans, compound heterozygous mutation of VPS51 results in global developmental delay, microcephaly, hypotonia, epilepsy, cortical vision impairment, pontocerebellar abnormalities, failure to thrive, liver dysfunction, lower extremity edema and dysmorphic features (Gershlick et al., 2019). Protein kinase LRRK2 involved in Parkinson disease is shown to interact with VPS52 to assist in membrane fusion at the TGN thereby acting as a scaffold between the GARP complex and SNAREs. Mutations in VPS52 can exacerbate Parkinson disease-associated toxicity (Beilina et al., 2020). A whole exome sequencing of the genomic DNA showed compound heterozygous mutation in VPS53 leading to Progressive cerebello-cerebral atrophy type 2 (PCCA2) (Feinstein et al., 2014). A missense mutation (L967Q) of VPS54 causing protein instability (Schmitt-John et al., 2005) was identified in the wobbler mouse, a model for motor neuron disease Amyotrophic lateral sclerosis (Pérez-Victoria et al., 2010; Moser et al., 2013). Moreover, a null mutation of VPS54 is embryonically lethal with high neural tube membrane blebbing phenotype (Karlsson et al., 2013).

Currently, it is not clear how disruption of the GARP complex function is associated with severe neurodevelopmental disorders. So, to bridge this gap in knowledge, it is very important to understand the functions of GARP. GARP was initially identified to have a role in sorting of Cathepsin D to lysosomes by assisting the retrieval of M6PR back to TGN (Pérez-Victoria et al., 2008). Another known function of GARP is in maintenance of sphingolipids, where defects in GARP complex result in the accumulation of sphingolipids synthesis intermediates and disruption in the distribution of sterol (Fröhlich et al., 2015). The GARP complex is also involved in intracellular cholesterol transport *via* targeting NPC2 to lysosomes (Wei et al., 2017). Auxin mediated depletion of GARP complex resulted in missorting of the flippases and remodeling of lipid composition in yeast (Eising et al., 2019). We have recently shown that a knock-out (KO) of the GARP complex subunits affects core Golgi function of *N*- and *O*- glycosylation as a result of reduction in total level of Golgi enzymes responsible for Golgi glycosylation (Khakurel et al., 2021).

In this study, we continue the investigation of the role of GARP in Golgi homeostasis by employing microscopy approaches and quantitative proteomics of isolated Golgi membranes and characterizing effects of GARP depletion on Golgi Ca^2+^ binding proteins, SNAREs and COPI vesicle budding machinery.

## Materials and methods

### Cell culture

hTERT RPE1 wild-type and GARP mutants were described previously (Khakurel et al., 2021). Cells were cultured in DMEM containing Nutrient mixture F-12 (Corning) supplemented with 10% fetal bovine serum (FBS) (Thermo Fisher) and incubated in a 37°C incubator with 5% CO2 and 90% humidity.

Plasmids encoding gRNAs targeted to GOSR1 (TEDH-1032327) or BET1L (TEDH-1007435) KOs were purchased from Transomics with the following target sequences:

#### GOSR1 (GS28)

1a) AAA​AGA​AAA​TAT​GAC​TTC​ACA​GAG​AGG​AAT
1b) AGC​GGC​GGG​ACT​CGC​TCA​TCC​TAG​GGG​GTG

#### BET1L (GS15)

1a) AAC​AGA​GAC​TCC​ATG​GTG​TTG​TGC​TGG​ACA
1b) AGA​CTA​TCA​TTC​CGG​ACG​TAG​ACG​TGG​CAC

Plasmids were isolated from bacteria using the QIAprep Spin Miniprep Kits (Qiagen). RPE1 cells stably expressing Cas9, described previously (Khakurel et al., 2021) were transfected by Neon electroporation (Thermo Fisher) according to the manufacturer’s protocol. To create GOSR1 (GS28KO) or BET1L (GS15KO), RPE1-Cas9 cells were transfected with the respective appropriate plasmid. After 16 h of transfection, untransfected cells were killed using 12 μg/ml Puromycin for 48 h. Surviving cells were then single cell plated on 96 well plates to obtain individual colonies depleted for the target protein. Knock-out was confirmed by western-blotting for the targeted protein.

### Cell fractionation

hTERT-RPE1 VPS54KO, VPS54KO R, VPS53KO, and VPS53KO R cells were plated in two 15 cm dishes in DMEM/F12 medium containing 10% FBS and grown to 100% confluence. Cells were washed twice with 10 ml of PBS and once with hypertonic 0.25 M sucrose in PBS. After the complete removal of PBS, the dishes were placed on ice and 1 ml of freshly prepared hypotonic ice-cold lysis buffer (20 mM HEPES pH 7.4, 1 mM PMSF, 5 μL/ml of HALT protease inhibitor) was added. The cells were collected with cell scrapper and moved to 1.5 ml microcentrifuge tube. Cells were lysed by passing through 26G syringe for 20 passages. The efficiency of lysis was examined by a phase contrast microscope equipped with 10x objective. Cell lysates were centrifuged at 3,000 g for 3 min at 4°C to pellet unlysed cells and nucleus. The S3 supernatant was transferred to Beckman 1.5 ml ultracentrifuge tube and subjected to centrifugation at 30,000 g for 30 min at 4°C. The supernatant (S30) was carefully removed without disturbing the pellet. The pellet (P30) was resuspended in 350 µL of 50% Nycodenz in the lysis buffer and transferred to 2.2 ml Beckman centrifuge tube on top of 100 µL of 60% Nycodenz. The sample was then overlaid with 35%, 30%, 25% and 20% Nycodenz (400 µL of each), and finally with 100 µL of lysis buffer. The gradient was centrifuged at 214000 g for 4 h at 4°C. 10 fractions (200 µL each) were collected from the top, mixed with 2x SDS sample buffer, heated at 70°C for 10 min and analyzed by western blot.

To prepare Golgi-enriched membranes for Mass spectrometric analysis, membranes floated in Nycodenz gradient (top 500 µL fraction) were collected, diluted with 1 ml of 20 mM HEPES with 150 mM NaCl and pelleted at 120000 g for 1 h at 4°C. The supernatant was removed, and the Golgi membrane pellets were submitted for label-free mass spectrometry analysis. Four biological replicates of each sample were analyzed.

### Mass-spectrometry and data analysis

Proteins were reduced, alkylated, and purified by chloroform/methanol extraction prior to digestion with sequencing grade modified porcine trypsin (Promega). Tryptic peptides were then separated by reverse phase XSelect CSH C18 2.5 um resin (Waters) on an in-line 150 × 0.075 mm column using an UltiMate 3000 RSLCnano system (Thermo). Peptides were eluted using a 60 min gradient from 98:2 to 65:35 buffer A:B ratio. Eluted peptides were ionized by electrospray (2.2 kV) followed by mass spectrometric analysis on an Orbitrap Exploris 480 mass spectrometer (Thermo). To assemble a chromatogram library, six gas-phase fractions were acquired on the Orbitrap Exploris with 4 m/z DIA spectra (4 m/z precursor isolation windows at 30,000 resolution, normalized AGC target 100%, maximum inject time 66 ms) using a staggered window pattern from narrow mass ranges using optimized window placements. Precursor spectra were acquired after each DIA duty cycle, spanning the m/z range of the gas-phase fraction (i.e., 496–602 m/z, 60,000 resolution, normalized AGC target 100%, maximum injection time 50 ms). For wide-window acquisitions, the Orbitrap Exploris was configured to acquire a precursor scan (385–1,015 m/z, 60,000 resolution, normalized AGC target 100%, maximum injection time 50 ms) followed by 50 × 12 m/z DIA spectra (12 m/z precursor isolation windows at 15,000 resolution, normalized AGC target 100%, maximum injection time 33 ms) using a staggered window pattern with optimized window placements. Precursor spectra were acquired after each DIA duty cycle.

Buffer A = 0.1% formic acid, 0.5% acetonitrile.

Buffer B = 0.1% formic acid, 99.9% acetonitrile.

Following data acquisition, data were searched using an empirically corrected library and a quantitative analysis was performed to obtain a comprehensive proteomic profile. Proteins were identified and quantified using EncyclopeDIA (Searle et al., 2018) and visualized with Scaffold DIA using 1% false discovery thresholds at both the protein and peptide level. Protein exclusive intensity values were assessed for quality using an in-house ProteiNorm app, a tool for systematic evaluation of normalization methods, imputation of missing values and comparisons of multiple differential abundance methods (Graw et al., 2020). Normalization methods evaluated included log2 normalization (Log2), median normalization (Median), mean normalization (Mean), variance stabilizing normalization (VSN) (Huber et al., 2002), quantile normalization (Quantile) (Bolstad, 2022), cyclic loess normalization (Cyclic Loess) (Ritchie et al., 2015), global robust linear regression normalization (RLR) (Chawade et al., 2014), and global intensity normalization (Global Intensity) (Chawade et al., 2014). The individual performance of each method was evaluated by comparing of the following metrices: total intensity, pooled intragroup coefficient of variation (PCV), pooled intragroup median absolute deviation (PMAD), pooled intragroup estimate of variance (PEV), intragroup correlation, sample correlation heatmap (Pearson), and log2-ratio distributions. The normalized data were used to perform statistical analysis using linear models for microarray data (limma) with empirical Bayes (eBayes) smoothing to the standard errors (Ritchie et al., 2015). Proteins with an FDR adjusted *p*-value <0.05 and a fold change >2 were considered significant.

### Western blot analysis

Total cell lysates were prepared as described before (Khakurel et al., 2021). Briefly, cells grown on tissue culture dishes were washed twice with PBS and lysed in 2% SDS preheated at 70°C. Cell lysates were collected and briefly sonicated to break chromosomal DNA. Protein concentration was measured using BCA protein assay (Pierce). 6× SDS sample buffer containing beta-mercaptoethanol was added and samples were heated at 70°C for 10 min. Samples (10–30 µg of protein) were loaded onto Bio-Rad (4%–15%) or Genescript (8%–16%) gradient gels. Proteins were transferred to nitrocellulose membranes using the Thermo Scientific Pierce G2 Fast Blotter. Membranes were washed in PBS, blocked in Odyssey blocking buffer (LI-COR) for 20 min, and incubated with primary antibodies for 1 h at room temperature (RT) or overnight at 4°C. Membranes were washed with PBS and incubated with secondary fluorescently tagged antibodies diluted in Odyssey blocking buffer for 60 min. All the primary and secondary antibodies used in the study are listed in Table 1. Blots were then washed and imaged using the Odyssey Imaging System. Images were processed using the LI-COR Image Studio software.

**TABLE 1:** List of Antibodies used in the study.

Antibody	Source/Catalog #	Species	WB dilution	IF dilution
Rab6 (C19)	Santa Cruz	Rabbit	—	1:400
	#SC310			
SDF4/Cab45	Sigma HPA011249	Rabbit	1:1000	1:400
ATP2C1	Proteintech	Rabbit	1:1000	—
	#I33I0-I-AP			
Golgin97	Invitrogen	Mouse	—	1:500
	#A21270			
GM130	BD Biosciences, 610823	Mouse	—	1:500
GM130	CalBiochem, CB1008	Rabbit	—	1:300
β-actin	Sigma, A5441	Mouse	1:1000	-
TGN46	Bio-Rad, AHP500G	Sheep	1:2000	-
GOSR1/GS28	BD Biosciences	Mouse	1:500	1:500
	#611184			
BET1L/GS15	BD Biosciences	Mouse	-	1:500
	#610961			
B4GALT1	R&D Systems, AF-3609	Goat	1:500	-
COPB1	Duden’s lab Duden et al. (1991)	Rabbit		1:2000
COPB2	ABclonal, A7036	Rabbit	—	1:400
COPG1	Duden’s lab	Rabbit	—	1:2000
ERGIC-53	Enzo OTI1A8	Mouse	—	1:2000
SNX1	BD	Mouse	—	1:300
	#S056			
GBF1	Sztul lab	Rabbit		1:300
GBF1	BD transduction laboratories	Mouse	—	1:500
	#612116			
BIG1(H-200)	Santa Cruz	Rabbit	—	1:300
	#sc-50391			
CD63	DSHB, H5C6-C	Mouse	—	1:200
LAMP2	DSHB, H4B4	Mouse	1:500	-
GOLPH3	Abcam	Rabbit	—	1:500
	# ab98023			
ARFGAP1	ABclonal	Rabbit	1:1000	1:500
	#A7118			
IRDye 680 anti-Mouse	LiCOR/926–68170	Goat	1:40000	-
IRDye 800 anti-Rabbit	LiCOR/926–32211	Goat	1:40000	-
IRDye 800 anti-Goat	LiCOR/926–32214	Donkey	1:40000	-
Alexa Fluor 647 anti-Rabbit	Jackson Immuno Research/711-605-152	Donkey	1:500	1:1000
Alexa Fluor 647 anti-Mouse	Jackson Immuno Research/715-605-151	Donkey	1:500	1:1000
Alexa Fluor 647 anti-Goat	Jackson Immuno Research/705-605-147	Donkey	—	1:1,000
DyLight 647 anti-Sheep	Jackson Immuno Research/713-605-147	Donkey	—	1:1000
anti-Rabbit Cy3	Jackson Immuno Research/711-165-152	Donkey	—	1:1000
anti-Mouse Cy3	Jackson Immuno Research/715-165-151	Donkey	—	1:1000
Alexa Fluor 488 anti-Rabbit	Jackson Immuno Research/711-545-152	Donkey	—	1:1000
Alexa Fluor 488 anti-Mouse	Jackson Immuno Research/715-545-151	Donkey	—	1:1000

### Airyscan microscopy

Cells were plated on round glass coverslips (12 mm diameter), grown to 80% confluence, washed with PBS and fixed with 4% paraformaldehyde (PFA) (freshly made from 16% stock solution) in PBS for 15 min at RT. Cells were permeabilized with 0.1% Triton X-100 for 1 min, blocked with 50 mM ammonium chloride for 5 min, washed with PBS, and incubated 2 times 10 min each in 1% BSA, 0.1% saponin in PBS. After that, cells were incubated with primary antibodies (diluted in 1% cold fish gelatin, 0.1% saponin in PBS) for 40 min, washed, and incubated with fluorescently conjugated secondary antibodies for 30 min. All the primary and secondary antibodies are listed in Table 1. Hoechst was used to stain chromosomal DNA. Cells were washed four times with PBS, then coverslips were dipped in PBS and water 10 times each and mounted on glass microscope slides using Prolong Gold antifade reagent (Life Technologies). Samples were imaged using 63× oil 1.4 numerical aperture objective with a LSM880 Airyscan Zeiss Laser inverted microscope. Quantitative analysis was performed using ZEN software or ImageJ on single-slice confocal images. Image processing including conversion of imaged z-stacks into maximum intensity-projections (MIPs) was done in ZEN software. Airyscan superresolution MIPs (6 z-stacks) were used in all Figures.

### Transmission electron microscopy

The samples were treated according to Valdivia’s protocol (Cocchiaro et al., 2008) with some modifications (Pokrovskaya et al., 2012). Briefly, RPE1 WT, VPS53KO, and VPS54KO cells were plated on a 6 well plate and once the cell reached 90% confluence, 1X fixative was added to equal volume of growing media and incubated at room temperature (RT) for 5 min. Next, 0.5X fixative was replaced with 1X fixative (composed of 4% Paraformaldehyde (EMS) and 1% Glutaraldehyde (GA) (EMS) in 0.1 M phosphate buffer of pH 7.4) and incubated for 15 min at RT. The cells were fixed with 2.5% GA, 0.05% malachite green (EMS) in 0.1 M sodium cacodylate buffer of pH 6.8. Cells were washed 4 times 5 min each with 0.1 M sodium cacodylate buffer and post-fixed with 0.5% osmium tetroxide and 0.8% potassium ferricyanide in 0.1 M sodium cacodylate buffer for 40 min at RT. The cells were washed again 4 times 5 min each. Then, the cells were incubated with 1% tannic acid for 20 min on ice, washed with buffer and then with water followed by incubation with 0.5% uranyl acetate (UA) at RT for 1 h. After washing with water, cells were scrapped off the plate and transferred to the tubes. 25%–100% gradual alcohol dehydration was done on ice. Cells were incubated in 100% alcohol 3 times 10 min each, in Propylene oxide (EMS) 3 times 10 min each, and in 1:1 mixture of Propylene oxide and Araldite 502/Embed 812 for overnight. Finally, samples were embedded in Araldite 502/Embed 812 resins (EMS) and hardened at 60°C for 48 h. Ultrathin sections were contrasted by Uranyl Acetate and Reynolds Lead Citrate stains and imaged at 80 kV on FEI Technai G2 TF20 transmission electron microscope. Digital images were acquired with FEI Eagle 4kX USB Digital Camera.

### Quantification of Golgi area

To analyze the *trans*- and *cis*-Golgi area, cells were stained for Rab6 and GM130 and imaged using Airyscan microscopy. Then ImageJ software was used to split the color channels on the individual confocal slices and create binaries. Next, under the function of “Analyze”, the measurement was set to “Area”. The scale of measurement was set for the Golgi particles with size of “2-Infinity” (microns^2) under the “Analyze particles” function. The Golgi “outlines” in both KO cells and control cells were noted and the graph was made using GraphPad Prism 9.3.0. At least 30 cells were used for quantification of Golgi area per group.

To determine the distance between the *trans*-Golgi and *cis*-Golgi in nocodazole-treated ministacks, a line was drawn in from the center of *cis*-Golgi to the center of *trans*-Golgi within each ministacks in Zen Blue Software. The distance between *cis* and *trans*-Golgi compartments was recorded (*n* = 30).

For the EM study, Golgi organization was analyzed ∼30 cells for each variant. The Golgi structure was assessed for three criteria: normal (tight, ribbon-like), swollen (severely expanded and vacuole-like) and fragmented stacks.

### Colocalization analysis

Pearson’s correlation coefficient was calculated using “Colocalization” module of Zen Blue (2.6) software. The colocalization between different proteins was recorded and the graph was made using GraphPad Prism 9.3.0. At least 30 cells were used for -slice and Pearson’s correlation coefficient was measured. The single-slice Airyscan images were used for the colocalization analysis.

### Statistical analysis

All the results are based on at least three biological experiments. WB images are representative from three repeats. WBs were quantified using the LI-COR Image Studio software. This study uses RPE1 VPS53KO R (rescue), VPS54KO R cells as a control, and the graph for WB displays fold change of the fluorescent band intensity in comparison to control. The control is considered as fold change of 1. The error bars for all graphs denote SD. At least 30 cells were used for statistical analysis of Airyscan microscopy. Statistical analysis was done using one-way ANOVA, or an unpaired *t* test in GraphPad Prism software.

### Controls used in the study

RPE1 VPS53KO R (rescue), VPS54KO R or WT cells are used as a control in the study. To avoid clonal variations, the rescued cells were not subcloned after stable re-expression of deleted subunits. The deleted subunits were re-expressed at the endogenous level. In our previous study (Khakurel et al., 2021), we have used both WT and KO R cells as a control and found consistent result with both of the controls. Proteomics analysis was done using VPS53KO R cells as a control for VPS53KO group and VPS54KO R cells as a control for VPS54KO group. For HEK293T and HeLa mutants, WT cells serve as a control.

## Results

### Depletion of GARP complex alters Golgi morphology

**FIGURE 1:**
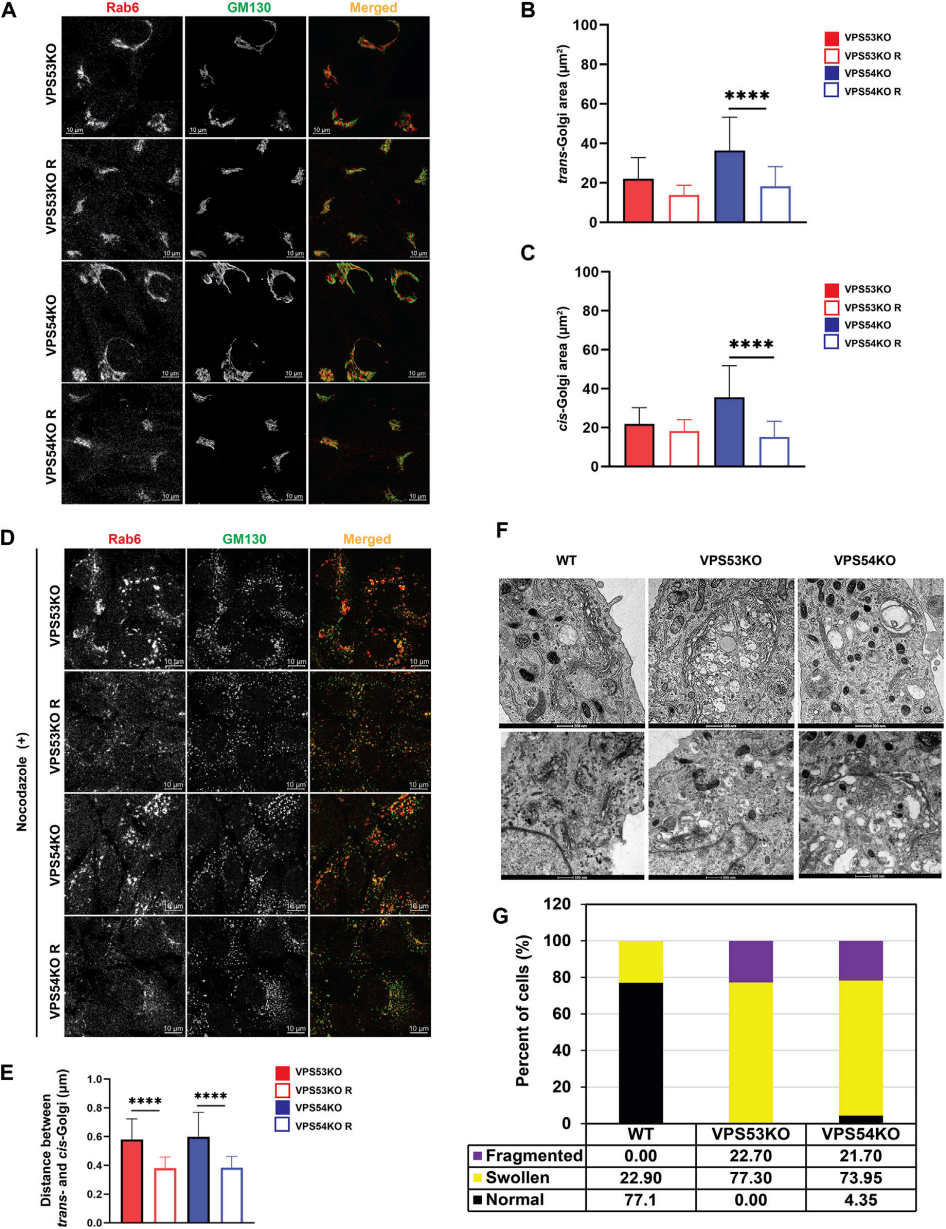
GARP deficiency affects Golgi structure. **(A)** Airyscan microscopy of VPS53KO, VPS53KO R, VPS54KO, and VPS54KO R cells stained for *trans*-Golgi Rab6 and *cis*-Golgi GM130. Individual channels are presented as black and white images, while overlays are presented as RGB images. **(B)** Quantification of *trans*-Golgi area. **(C)** Quantification of *cis*-Golgi area. *n* = 30 cells per group. **(D)** Airyscan microscopy of Nocodazole treated cells, stained for *trans*-Golgi Rab6 and *cis*-Golgi GM130. **(E)** Quantification of the distance between the *cis*- (GM130) and *trans*- (Rab6) Golgi compartments. **(F)** Transmission electron microscopy of WT, VPS53KO, and VPS54KO RPE1 cells. **(G)** Quantification of normal and abnormal (swollen or fragmented) Golgi phenotypes in WT, VPS53KO, and VPS54KO cells. *n* = 30 cells were imaged per group for the quantification. Statistical significance was calculated using one-way ANOVA in GraphPad prism. **** *p* ≤ 0.0001.

Upon microscopic analysis of GARP-deficient RPE1 cells (Khakurel et al., 2021), we noticed that Golgi structures in mutant cells looked enlarged and morphologically different from wild type cells. To test if GARP complex subunits knock-out (KO) cells have a significant alteration in Golgi size, we stained them using antibodies to *cis*-Golgi protein GM130 and *trans*-Golgi marker Rab6 and examined the size of Golgi compartments using Airyscan superresolution microscopy (Figure 1A). Both *trans*- and *cis*-Golgi areas were significantly increased in VPS54KO cells, while the Golgi increase in VPS53KO cells was less dramatic (Figures 1B, C). Follow-up analysis revealed a significant expansion of the Golgin-97 labeled TGN in both mutants (Figure 3C). To further investigate Golgi changes in GARP-KO cells, we treated them with Nocodazole to disperse Golgi ribbon into ministacks (Minin, 1997; Yang and Storrie, 1998). This approach allowed us to obtain more accurate information about the “Golgi thickness” - the distance between *cis*- and *trans*-Golgi compartments (Figure 1D). As expected, Rab6 stained mini-Golgi appeared larger in KO cells and Golgi thickness was significantly increased in both VPS54 and VPS53 deficient cells (Figure 1E). To complement the results obtained with Airyscan microscopy, we analyzed GARP-KO cells by Transmission Electron Microscopy (TEM). TEM analysis revealed a tight Golgi ribbon in wild-type (WT) cells whereas, in VPS53KO and VPS54KO cells, one side of the Golgi complex was severely swollen, and the entire Golgi was more fragmented (Figures 1F, G). Large vacuolar structures observed in GARP-KO cells are likely to represent swollen TGN and/or enlarged endolysosomal compartments.

### Proteomic analysis revealed depletion of a subset of Golgi proteins in GARP-KO cells

**FIGURE 2:**
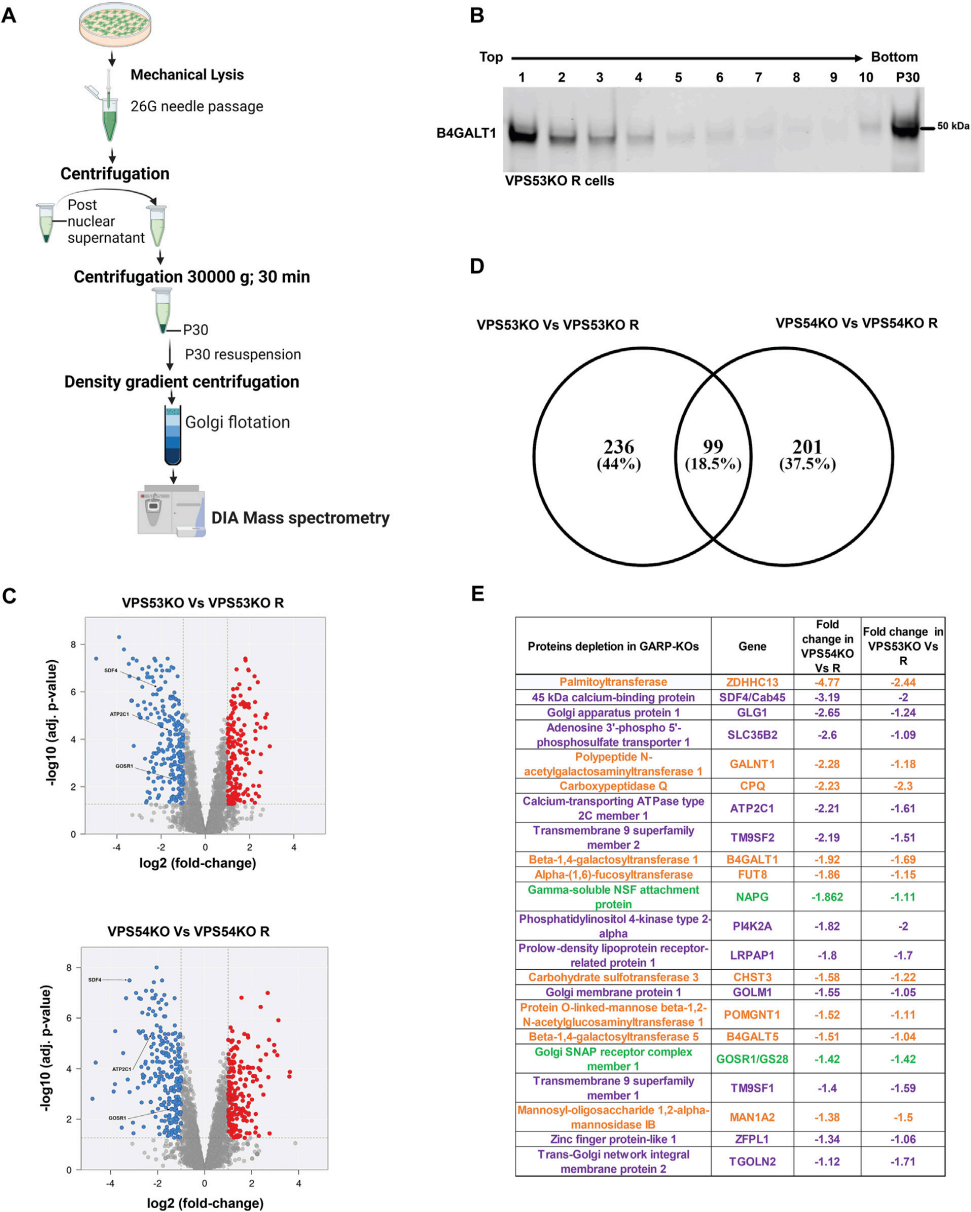
Quantitative Mass spectrometry (MS) analysis of Golgi-enriched membranes isolated from GARP-KOs revealed depletion of multiple Golgi proteins. **(A)** Schematic of the major steps during preparation of Golgi samples for MS analysis. Schematic was made in BioRender. **(B)** WB for Golgi marker B4GALT1 in membranes isolated from VPS53KO R cells and separated by the Nycodenz flotation gradient. **(C)** Volcano plot analysis of relative protein abundance in VPS53KO and VPS53KO R (Top) and VPS54KO and its VPS54KO R cells (Bottom). Blue circles represent the proteins significantly depleted in GARP-KO cells. Red circles represent proteins significantly increased in GARP-KO cells. Gray circles are proteins with no significant differences. **(D)** Venn diagram demonstrating common proteins depleted in both VPS53KO and VPS54KO cells. **(E)** List of Golgi proteins significantly depleted in both VPS53KO and VPS54KO cells. Proteins with color orange are Golgi enzymes, purple are Golgi resident proteins, and Green are Golgi SNAREs.

Altered Golgi morphology in GARP-KO cells as well as our previous finding that GARP-KO cells are deficient in several components of Golgi glycosylation machinery (Khakurel et al., 2021), triggered the obvious question - how many Golgi proteins depend on the GARP complex for their localization and expression? To answer this question, we utilized label-free protein mass spectrometry proteomic analysis (MS) and compared the abundance of Golgi resident proteins in VPS53KO, VPS54KO, and control cells (Figure 2A). For Golgi isolation, control and KO cells were mechanically lysed and fractionated by differential centrifugation to obtain Golgi-enriched 30 K membrane pellet. Golgi membranes were further purified from cytoplasmic proteins by floating in the 20%–35% Nycodenz density gradient. After centrifugation, gradient fractions were collected for WB analysis. Golgi resident protein B4GALT1 was used as a marker for the Golgi membranes and the majority of B4GALT1 was found in fractions 1-3 both in VPS53KO R cells (Figure 2B) and all other WT and COG-deficient cells (A.K. & V.L. unpublished data). Top fractions were also enriched for Golgi proteins STX5 and TMEM165, while the mitochondrial marker COX4 was found in fractions 3–6 (A.K. & V.L. unpublished data). Fractions enriched for B4GALT1 were used for quantitative MS analysis. The MS analysis revealed a significant depletion of 335 proteins in VPS53KO cells, whereas there were 300 proteins depleted in VPS54KO cells (Figures 2C, D). There were 99 common proteins that were depleted in both VPS53KO and VPS54KO cells (Figure 2D). Of these 99 proteins, 22 were Golgi proteins (Figure 2E) represented by glycosylation enzymes (orange label), fusion machineries (green), and other Golgi resident proteins (purple). Our previous work (Khakurel et al., 2021) demonstrated that two of these proteins, B4GALT1 and TGN46 are severely depleted in GARP-KO cells. While most GARP-dependent proteins localize in *trans*-Golgi compartments, some of them, like GLG1/MG-160, GALNT1, MAN1A2 (Gonatas et al., 1989; Sahu et al., 2022), are reported to localize in *cis/medial* Golgi cisternae, indicating that GARP dysfunction is affecting the entire Golgi complex.

### GARP dysfunction affects Golgi calcium pump and calcium binding protein SDF4

**FIGURE 3:**
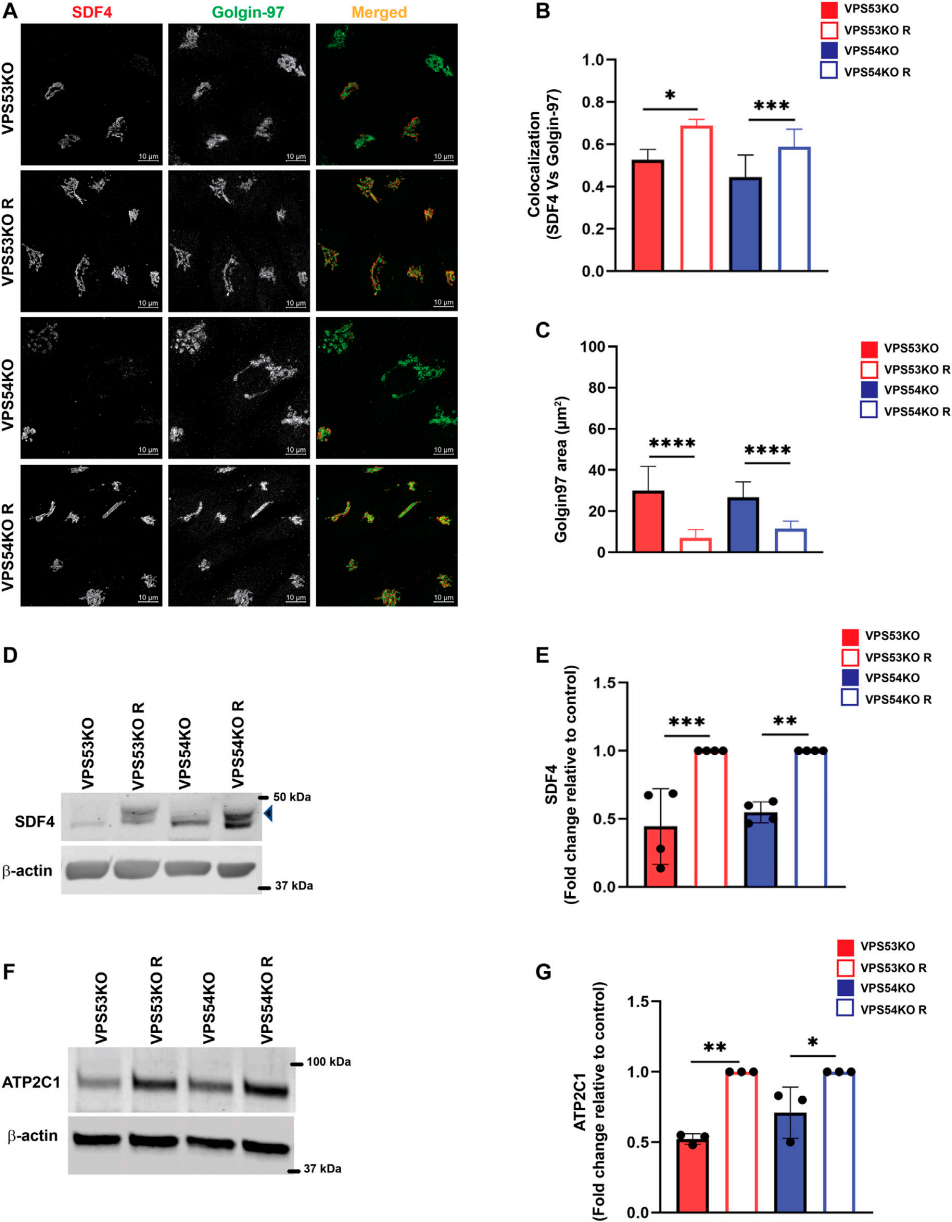
GARP dysfunction alters cellular expression of Ca^2+^ binding SDF4/Cab-45 and Ca^2+^-transporting ATP2C1/SPCA1 Golgi proteins. **(A)** Airyscan microscopy of VPS53KO, VPS53KO R, VPS54KO and VPS54KO R cells co-stained for SDF4 and Golgin97. **(B)** The graph shows the quantification of SDF4 and Golgin97 colocalization using Pearson’s correlation coefficient. Values in the bar graph represent the mean ± SD from the colocalization between SDF4 and Golgin97 from 50 different cells. **(C)** Quantification of Golgin97 area. *n* = 30 cells per group. **(D)** WB analysis of SDF4 in GARP-KO and rescued cells. The arrowhead pointing to SDF4 is the specific band. **(E)** Quantification of SDF4 WBs from four independent experiments. **(F)** WB analysis of ATP2C1 in GARP-KO and rescued cells. **(G)** Quantification of ATP2C1 blots from three independent experiments. β-actin was used as the internal loading control. Values in bar graph represent the mean ± SD from at least three independent experiments. Statistical significance was calculated using one-way ANOVA. *** *p* ≤ 0.001, ** *p* ≤ 0.01, * *p* ≤ 0.05.

To validate MS results, we tested the localization and abundance of several Golgi proteins, first focusing on two key players in Golgi Ca^2+^ homeostasis, SDF4/Cab45 and ATP2C1/SPCA1. SDF4 is a *trans*-Golgi network luminal calcium-binding protein that promotes sorting of a subset of secretory proteins at the *trans*-Golgi Network (TGN) (Blank and von Blume, 2017). To verify the SDF4 MS results, we co-stained VPS53KO, VPS54KO and control cells with SDF4 and a GARP-independent *trans*-Golgi protein Golgin97/GOLGA1 (Ishida and Bonifacino, 2019) (Figure 3A). Airyscan microscopy revealed a significant decrease in SDF4 signal intensity at the Golgi and a decreased colocalization of SDF4 with Golgin97 supporting MS results (Figure 3B). Furthermore, while the total SDF4 protein level was similar between wild-type and rescued cells (A.K. unpublished data), SDF4 was significantly decreased in both VPS53KO and VPS54KO cells (Figures 3D, E). Ca^2+/^Mn^2+^ ATPase ATP2C1/SPCA1 (von Blume et al., 2011; Kienzle et al., 2014) is another Golgi protein that was depleted in the Golgi membranes isolated from GARP-KOs. Like in the case of SDF4, we found a significant reduction in the total protein abundance of ATP2C1 in GARP-KOs (Figures 3F, G). In summary, our analysis revealed two proteins related to Ca^2+^ Golgi homeostasis are depleted in GARP-KO cells.

### GARP depletion alters expression and localization of intra-Golgi v-SNAREs

**FIGURE 4:**
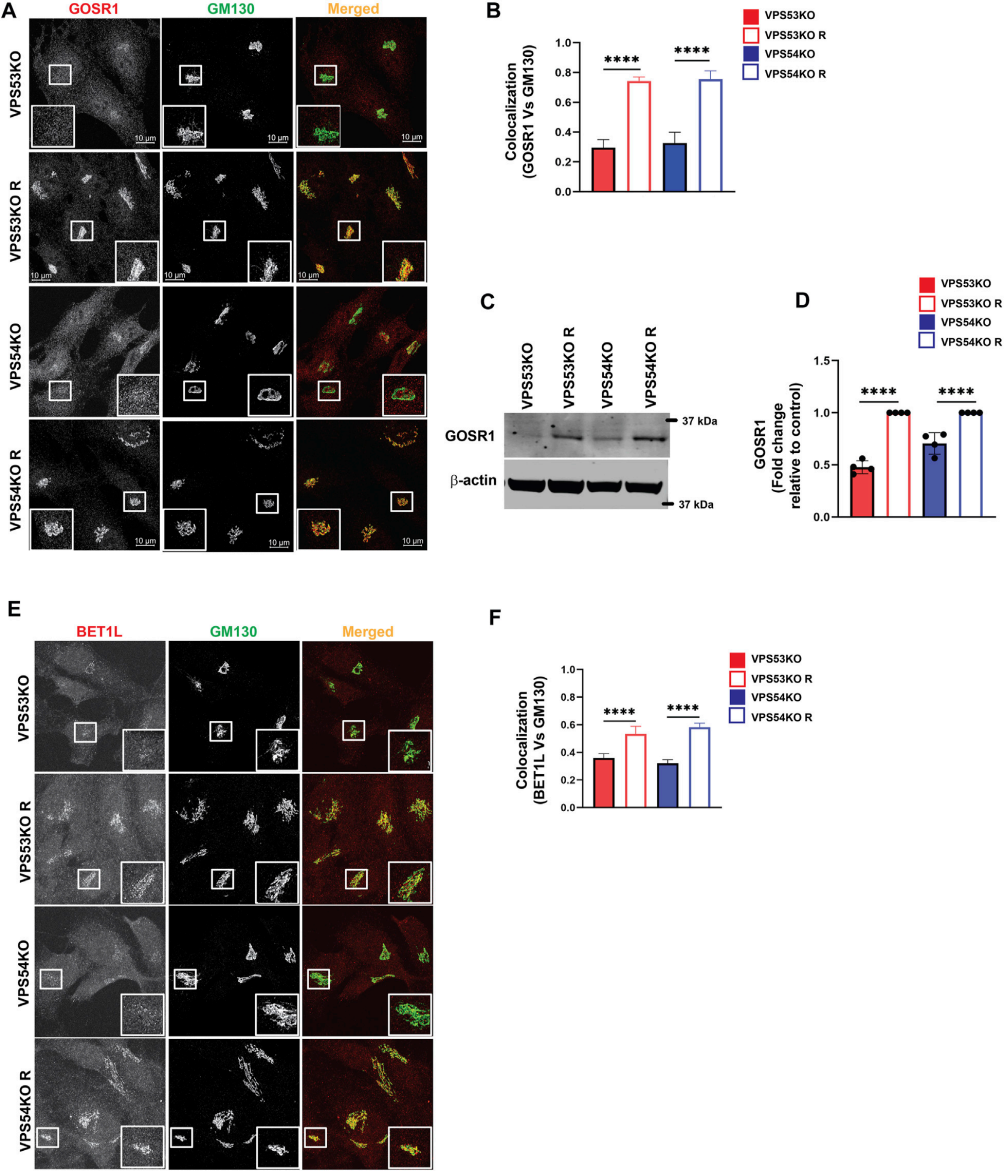
GARP-KO alters localization and expression of intra-Golgi v-SNAREs GOSR1 and BET1L. **(A)** Airyscan microscopy of GARP-KOs and control cells co-stained for GOSR1 and GM130. The large white square box inside the image is the zoomed view of the small square box (2X inset). **(B)** The graph shows the quantification of GOSR1 and GM130 colocalization. Values in the bar graph represent the mean ± SD from the colocalization between GOSR1 and GM130 from 40 different cells. **(C)** WB analysis of GOSR1 protein level in VPS53KO, VPS54KO, and their controls. β-actin was used as internal loading control. **(D)** Quantification of the blots from four independent experiments. **(E)** Airyscan imaging of VPS53KO, VPS53KO R, VPS54KO, VPS54KO R cells co-stained for BET1L and GM130. The large white square box inside the image is the zoomed view of the small square box (2X inset). **(F)** Colocalization analysis between BET1L and GM130 using Pearson’s correlation coefficient. *n* = 40 cells were imaged per group for the quantification. Statistical significance was calculated using one-way ANOVA. **** *p* ≤ 0.0001.

**FIGURE 5:**
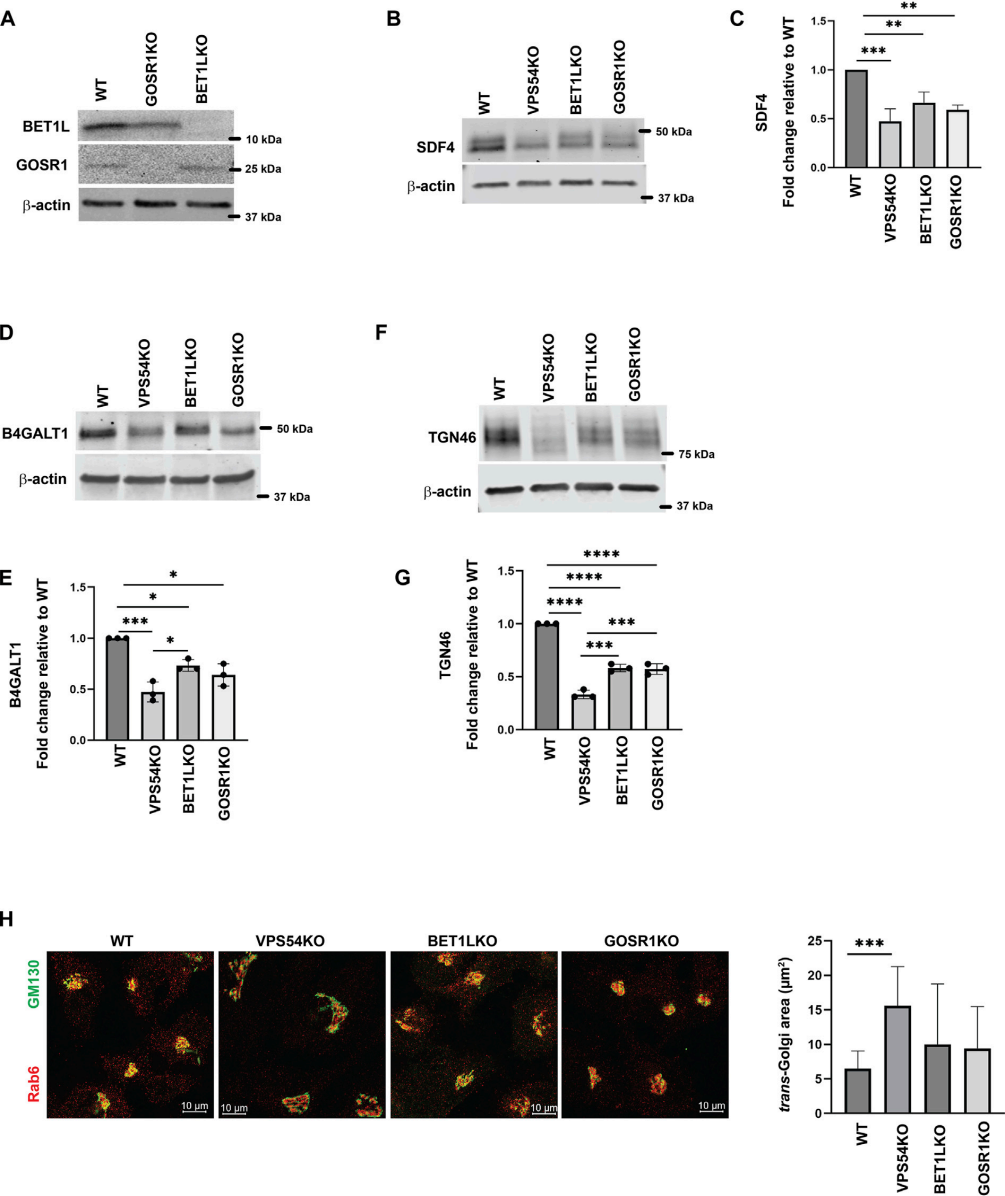
GARP-KO is more detrimental to Golgi residents than deletion of Golgi v-SNAREs. **(A)** Testing of GOSR1KO and BET1LKO by WB analysis. **(B)** Total cellular lysates were prepared from RPE1 WT, VPS54KO, BET1LKO, and GOSR1KO cells and total protein abundance was analyzed by WB for SDF4, **(D)** B4GALT1, and **(F)** TGN46. Normalization was performed using β-actin. Quantification of relative total protein level of **(C)** SDF4, **(E)** B4GALT1, and **(G)** TGN46. Statistical analysis was done from three independent blots, where **** *p* ≤ 0.0001, *** *p* ≤ 0.001, ** *p* ≤ 0.01, * *p* ≤ 0.05. **(H)** (Left panel) Airyscan microscopy of RPE1 WT, VPS54KO, BET1LKO, and GOSR1KO cells stained for Rab6 and GM130. (Right panel) Quantification of *trans*-Golgi Rab6 area in WT, VPS54KO, BET1LKO, and GOSR1KO.

Another key component of Golgi homeostasis significantly depleted in the GARP-deficient Golgi was Qb-SNARE GOSR1/GS28. We validated the MS results both by IF (Figures 4A, B) and WB analysis (Figures 4C, D). GOSR1 was mostly localized in the Golgi in control cells and was displaced from GM130-defined Golgi region in VPS53KO and VPS54KO cells confirming that the intracellular localization of this Golgi SNARE is severely altered in GARP-KOs. We next tested the total protein level of GOSR1 and observed a significant depletion in the abundance of GOSR1 in total cell lysates of GARP-KOs (Figures 4C, D). GOSR1 works in the STX5/GOSR1/BET1L/YKT6 SNARE complex to facilitate fusion of intra-Golgi recycling vesicles (Linders et al., 2019). We have previously shown that Qc-SNARE, BET1L/GS15, is depleted in GARP-KO cells (Khakurel et al., 2021). Like GOSR1 results, severe mislocalization of BET1L was observed in both VPS54KO and VPS53KO cells (Figures 4E, F). Proteomics data indicated that two other SNAREs of this complex, YKT6 and STX5, are not depleted in the Golgi-enriched membranes from GARP mutants, indicating that GARP deficiency only affects putative v-SNARE proteins. This is in line with the data obtained in COG-deficient cells (Zolov and Lupashin, 2005). GOSR1 and BET1L are two major Golgi Q-SNAREs involved in the intra-Golgi retrograde trafficking of Golgi resident proteins (Linders et al., 2019) and their mislocalization and depletion could be responsible for Golgi defects in GARP-KO cells. To test this possibility, we created RPE1 cells deleted for GOSR1 and BET1L (Figure 5A). Deletion of these Golgi SNAREs did not alter RPE1 cell growth (data not shown), similar to SNARE KO results in HEK293T cells (D'Souza et al., 2022). To test if SNARE depletion could compromise the total protein level of Golgi resident proteins, the total protein abundance of SDF4, B4GALT1 and TGN46 was compared between WT, SNAREs-KO (BET1LKO, GOSR1KO) and GARP-KO (VPS54KO) cells (Figures 5B, C). Although the abundance of SDF4 (Figures 5B, C), B4GALT1 (Figures 5D, E) and TGN46/TGOLN2 (Figures 5F, G) was reduced in SNARE-KO cells, GARP depletion demonstrated a greater decrease in the abundance on all three Golgi resident proteins, indicating that depletion of Golgi SNAREs alone could not explain Golgi defects in GARP-KO cells (Figures 5B–G). Another important phenotype observed in GARP-KO cells is their alteration in Golgi size as shown in Figure 1A. We, therefore, examined Golgi morphology by staining the WT, BET1LKO, GOSR1KO and VPS54KO cells with *trans*-Golgi marker Rab6 and *cis*-Golgi marker GM130 (Figure 5H). Airyscan microscopy analysis revealed that the Golgi apparatus was more enlarged in VPS54KO cells compared to SNARE-KO cells confirming that GARP-KO Golgi-related defects are more severe than defects observed in SNAREs-KOs.

### COPI vesicle coat proteins relocate to ERGIC in GARP-KO cells

**FIGURE 6:**
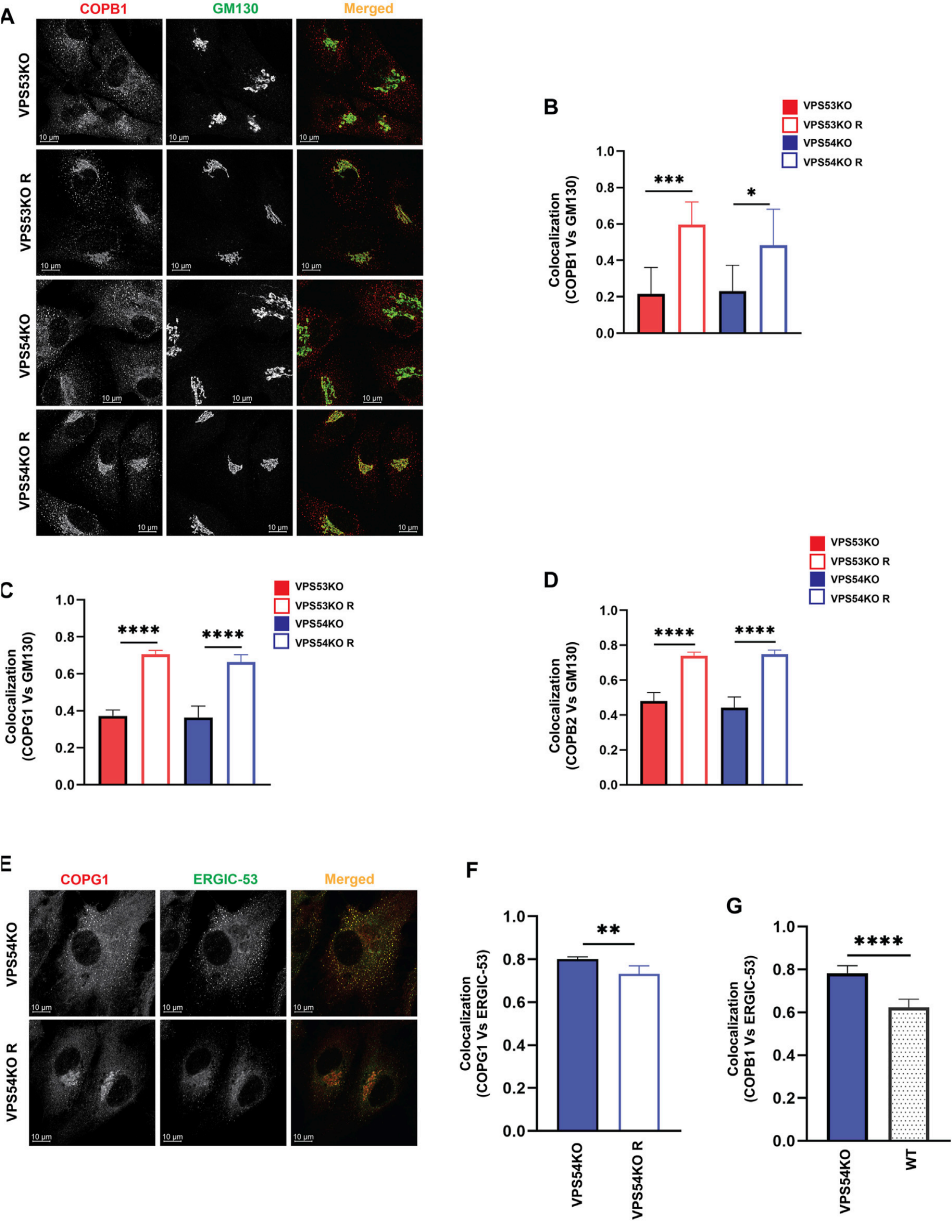
COPI subunits are displaced from the Golgi in GARP-KO cells. **(A)** Airyscan microscopy of GARP-KO cells and control cells stained with antibody to coatomer subunit COPB1. Colocalization analysis of COPB1 and GM130 **(B)**, COPG1 and GM130 **(C)**, COPB2 and GM130 **(D)** using Pearson’s correlation coefficient. n = 40 cells used for colocalization analysis per group. **(E)** Airyscan microscopy of COPG1 and ERGIC-53 in VPS54KO and VPS54KO R cells. **(F)** Colocalization analysis of COPG1 and ERGIC-53 and **(G)** COPB1 and ERGIC-53 using Pearson’s correlation coefficient. *n* = 40 cells used for colocalization analysis. Statistical significance was calculated using one-way ANOVA. **** *p* ≤ 0.0001, ** *p* ≤ 0.01.

Severe depletion of intra-Golgi trafficking SNAREs GOSR1 and BET1L in GARP-KO cells should lead to defects in vesicle fusion-the final step in vesicular trafficking but TEM analysis of GARP-KO cells did not reveal any significant accumulation of vesicular structures (Figure 1F), suggesting that formation of transport vesicles in mutant cells could be altered as well. To test this possibility, we analyzed the localization of vesicle coat machinery in GARP-KO cells. COPI complex consists of seven subunits - beta, gamma, delta, zeta, alpha, beta prime and epsilon (Hara-Kuge et al., 1994). We performed IF and investigated both proximal membrane coat proteins (COPB1, COPG1) as well as distal membrane coat subunit COPB2. We observed a significant reduction in juxtanuclear localization of both layers of COPI coat (Figures 6A–D) in GARP-KOs, with the majority of COPI staining appearing as peripheral dots. To determine if COPI is relocalized to pre- or post-Golgi compartments, we co-stained cells with COPI subunits and ERGIC-53 and found their colocalization increased in VPS54KO cells (Figures 6E–G). Co-staining with COPG1 and SNX1 did not reveal any significant colocalization between COPI and endosomes (A.K. unpublished data).

### GARP dysfunction leads to mislocalization of COPI accessory proteins

**FIGURE 7:**
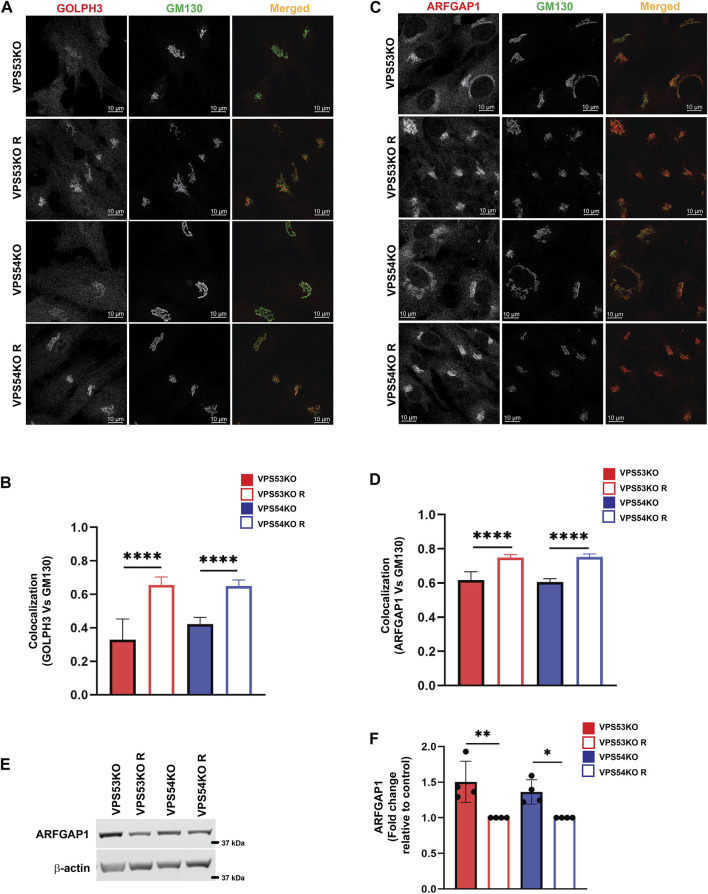
GARP dysfunction resulted in mislocalization of COPI adapter protein GOLPH3 and ARFGAP1 from the Golgi. **(A)** Airyscan microscopy of VPS53KO, VPS53KO R, VPS54KO, VPS54KO R cells stained with GOLPH3 and GM130. **(B)** Colocalization analysis between GOLPH3 and GM130 using Pearson’s correlation coefficient. *n* = 40 cells were used for colocalization analysis. **(C)** VPS53KO, VPS53KO R, VPS54KO, and VPS54KO R cells were stained for ARFGAP1 and GM130. **(D)** Colocalization analysis of ARFGAP1 and GM130 using Pearson’s correlation coefficient. *n*= 40 cells were used for colocalization analysis. **(E)** WB analysis of ARFGAP1 total protein abundance in GARP-KO and control cells. **(F)** Quantification of total protein abundance of ARFGAP1 in GARP-KOs and control cells. β-actin was used as internal loading control. **** *p* ≤ 0.0001, ** *p* ≤ 0.01, * *p* ≤ 0.05.

**FIGURE 8:**
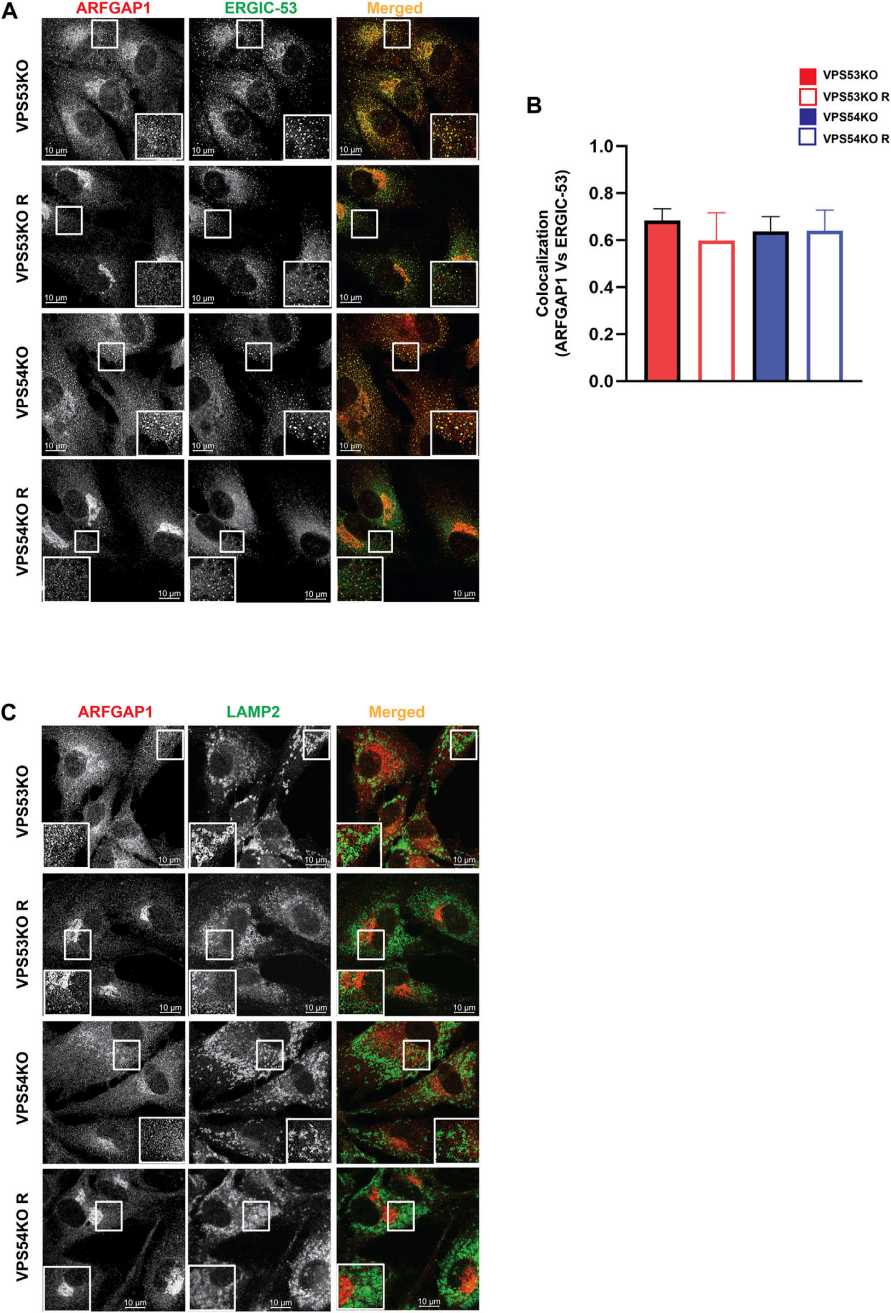
GARP depletion does not affect ARFGAP1 localization to ERGIC. **(A)** Airyscan microscopy of GARP-KO and control cells co-stained for ARFGAP1 and ERGIC-53. The large white square box inside the image is the zoomed view of the small square box (2X inset). **(B)** Colocalization analysis of ARFGAP1 and ERGIC-53 using Pearson’s correlation coefficient. **(C)** Airyscan microscopy of GARP-KO and control cells stained for ARFGAP1 and LAMP2. The large white square box inside the image is the zoomed view of the small square box (2X inset).

Our previous study (Khakurel et al., 2021) and the current proteomics data revealed depletion of several Golgi glycosyltransferases (B4GALT1, GALNT1, ST6GAL1 and MGAT1) in GARP-KO cells. GOLPH3 plays a crucial role in retrograde intra-Golgi trafficking of glycosyltransferases (Frappaolo et al., 2020). It is shown to bind the cytoplasmic tails of Golgi enzymes and packages them into recycling COPI vesicles (Welch et al., 2021). ARFGAP1 promotes the formation of COPI vesicles (Yang et al., 2002; Shiba and Randazzo, 2012). Both GOLPH3 and ARFGAP1 are peripheral membrane proteins that were stripped from membranes during flotation in the Nycodenz gradient and not detected by MS analysis in the Golgi-enriched membranes. But since the localization of COPI was found to be GARP-sensitive, the localization of GOLPH3 and ARFGAP1 in GARP deficient cells was tested. We found a decrease in Golgi localization of both GOLPH3 (Figures 7A, B) and ARFGAPs (Figures 7C, D), indicating that COPI accessory proteins also require GARP activity for their proper localization. However, unlike intra-Golgi v-SNAREs, the total cellular level of ARFGAP1 protein was increased (Figures 7E, F), while GOLPH3 expression was not altered (A.K. unpublished data) in GARP-KO cells. Since ARFGAP1 is present on both Golgi and ERGIC (ER-Golgi intermediate compartment) (Saitoh et al., 2009), we investigated possible displacement of ARFGAP1 from the ERGIC (Figure 8A). In agreement with COPI behavior, colocalization of ARFGAP1 with ERGIC-53 was found unchanged or slightly increased in GARP-KO cells (Figure 8B). ARFGAP1 did not show any colocalization with LAMP2 in both control and mutant cells (Figure 8C), indicating that GARP depletion resulted in specific displacement of both COPI and ARFGAP1 from the Golgi to the ERGIC.

### Localization of ARFGEFs is severely affected in GARP-KO cells

**FIGURE 9:**
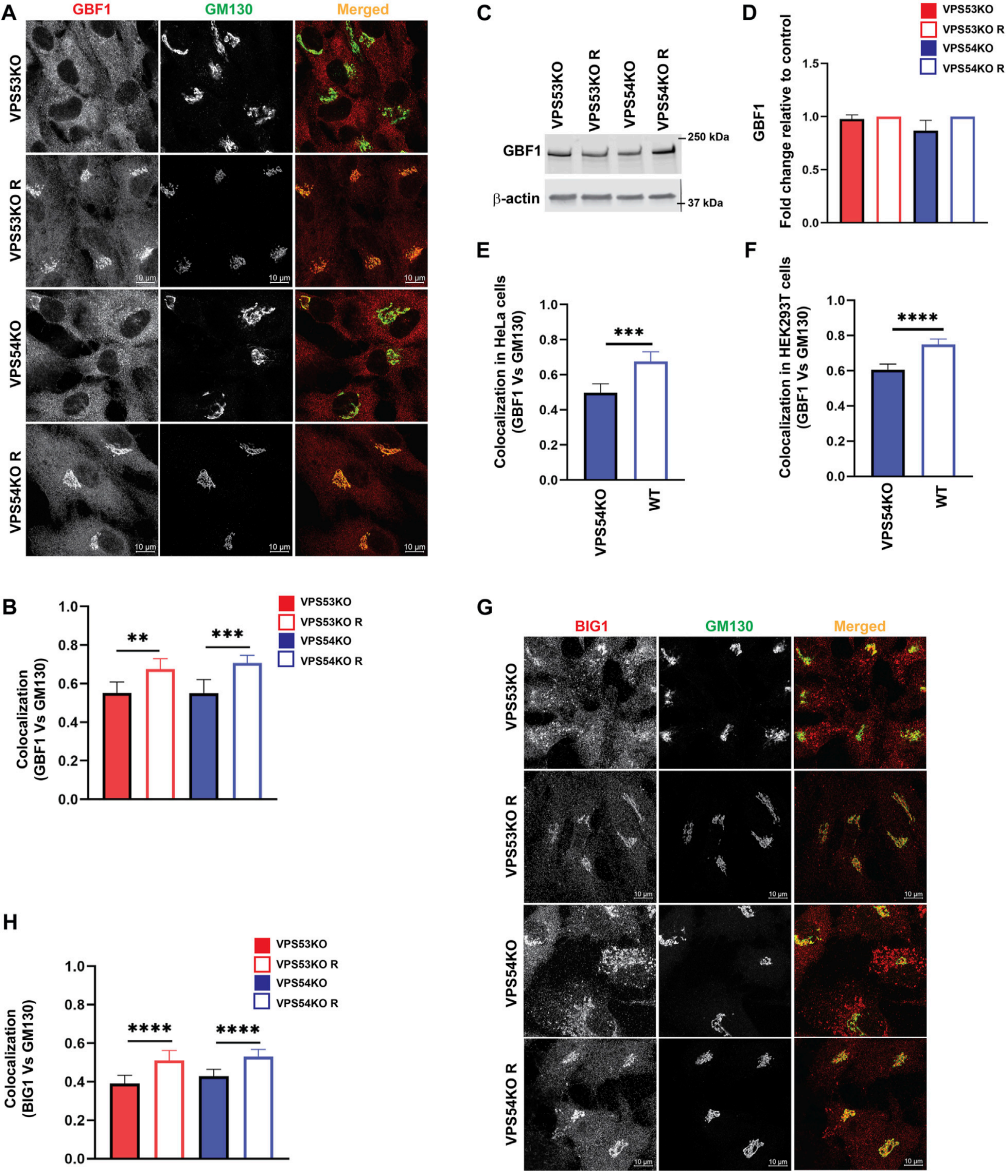
ARFGEFs GBF1 and BIG1 are displaced to off-Golgi compartments in GARP-KO cells. **(A)** Airyscan microscopy of GARP-KO and control cells co-stained with antibodies to GBF1 and GM130 in RPE1 cells. **(B)** Colocalization analysis of GBF1 and GM130 in RPE1 cells. **(C)** WB analysis of GBF1 total protein abundance in GARP-KO and control cells. **(D)** Quantification of total protein abundance of GBF1 in GARP-KOs and control cells. β-actin was used as the internal loading control. **(E)** Colocalization analysis of GBF1 and GM130 in HeLa cells. **(F)** Colocalization analysis of GBF1 and GM130 in HEK293T cells. **(G)** Airyscan microscopy of GARP-KO and control cells co-stained with antibodies to BIG1 and GM130 in RPE1 cells. **(H)** Colocalization analysis of BIG1 and GM130 using Pearson’s correlation coefficient. *n* = 50 cells were used for colocalization analysis. **** *p* ≤ 0.0001, *** *p* ≤ 0.001, **p ≤ 0.01.

**FIGURE 10:**
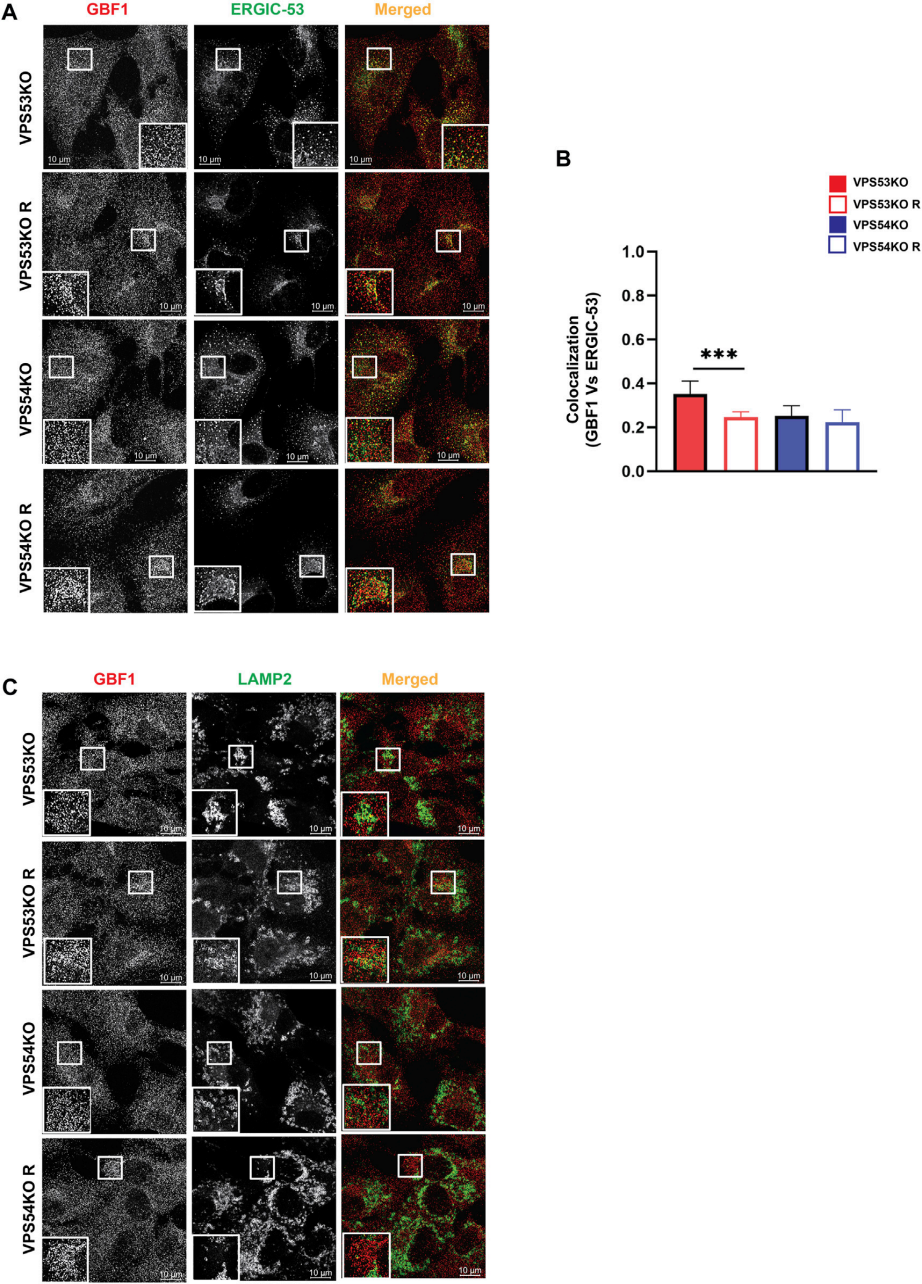
GBF1 is partially relocalized to ERGIC in GARP-KO cells. **(A)** Airyscan microscopy of VPS53KO, VPS54KO and their rescued cells for GBF1 and ERGIC-53. **(B)** Colocalization analysis of GBF1 and ERGIC-53 using Pearson’s correlation coefficient. **(C)** Airyscan microscopy of VPS53KO, VPS54KO and their rescued cells for GBF1 and LAMP2.

COPI binding to Golgi membrane requires activation of ARF GTPases, which is facilitated by ARFGEF proteins GBF1, BIG1/ARFGEF1, and BIG2/ARFGEF2 (Donaldson and Jackson, 2011). GBF1 is an ARFGEF found in *cis*-Golgi and ERGIC (Sztul et al., 2019). To test if GARP-KOs have any effect on *cis*-ARFGEF that could prevent COPI assembly at the Golgi, we stained cells with GBF1 and *cis*-Golgi marker GM130 (Figure 9A). Interestingly, we found a significant displacement of GBF1 from the *cis*-Golgi in GARP-KO cells (Figure 9B). However, the total level of GBF1 protein was not affected (Figures 9C, D). GBF1 phenotype was not cell-line dependent as both HeLa (Figure 9E) and HEK293T (Figure 9F) GARP-depleted cells showed a decrease in colocalization of GBF1 with GM130. We next investigated if GBF1, like COPI and ARFGAP1, is relocalized to ERGIC (Figures 10A, B). Indeed, we found that in VPS53KO cells colocalization between GBF1 and ERGIC-53 was significantly increased. Again, no colocalization between GBF1 and LAMP2 was observed in both mutant and rescued cells (Figure 10C), indicating that in GARP-deficient cells GBF1 is behaving similarly to COPI and ARFGAP1.

**FIGURE 11:**
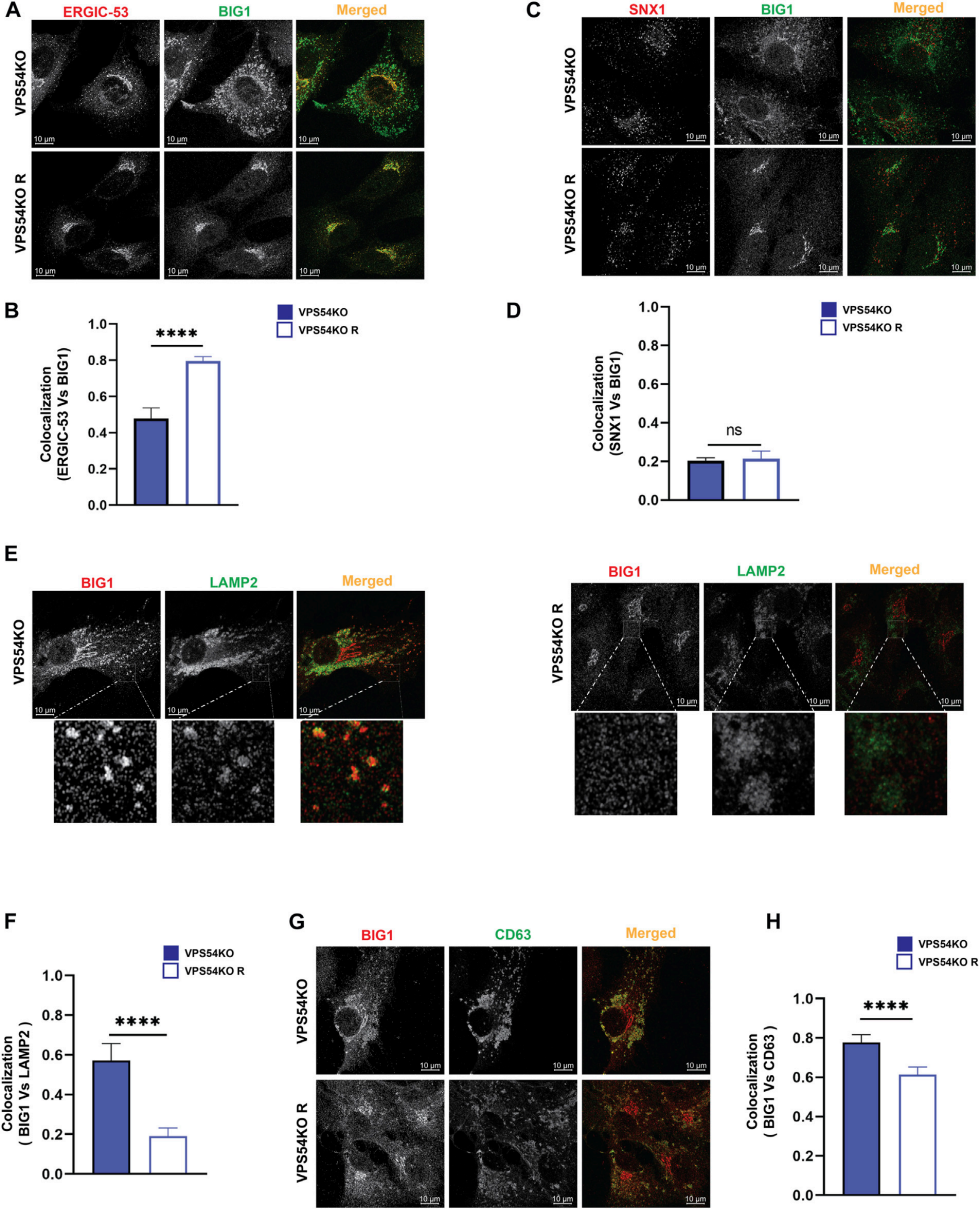
BIG1 is mislocalized to endolysosomal compartment in GARP-KOs. Airyscan microscopy of VPS54KO and VPS54KO R cells co-stained for BIG1 and ERGIC-53 **(A)**, BIG1 and SNX1 **(C)**, BIG1 and LAMP2 **(E)**, BIG1 and CD63 **(G)**. The small square box in **(E)** left and right panel indicates enlarged images. Colocalization analysis between BIG1 and ERGIC-53 **(B)**, BIG1 and SNX1 **(D)**, BIG1 and LAMP2 **(F)**, BIG1 and CD63 **(H)** by Pearson’s correlation coefficient analysis. *n* = 30 cells. **** *p* ≤ 0.0001, ** *p* ≤ 0.01.

Investigation of *trans*-Golgi ARFGEF BIG1 also revealed GARP-dependent mislocalization (Figures 9G, H), with a punctate BIG1 pattern in GARP-KO cells. Since BIG1 functions at *trans*-Golgi and endosomes (Christis and Munro, 2012) we have tested for potential relocalization of this ARFGEF to both ERGIC and post-Golgi compartments. Co-staining cells with BIG1 and ERGIC-53 (Figures 11A, B) or with BIG1 and SNX1 (Figures 11C, D) did not reveal any significant increase in colocalization, indicating that BIG1 did not relocalize to the ERGIC or sorting endosomes. In fact, BIG1 colocalization with ERGIC-53 on the Golgi was significantly decreased (Figures 11A, B). Furthermore, we tested if BIG1 is localized to LAMP2 compartment in GARP-KOs (Figure 11E). Surprisingly, we found a significant increase in colocalization of BIG1 to LAMP2 compartment in GARP-KOs (Figure 11F). Similar results were obtained by co-staining cells with BIG1 and another marker of endolysosomal compartment, CD63 (Figure 11G). Again, a significant increase in colocalization between CD63 and BIG1 was observed (Figure 11H). Taken together, these results indicate that GARP depletion affects Golgi localization of ARFGEFs GBF1 and BIG1, and BIG1, displacing GBF1 to the ERGIC and BIG1 to endolysosomal compartments.

## Discussion

**FIGURE 12:**
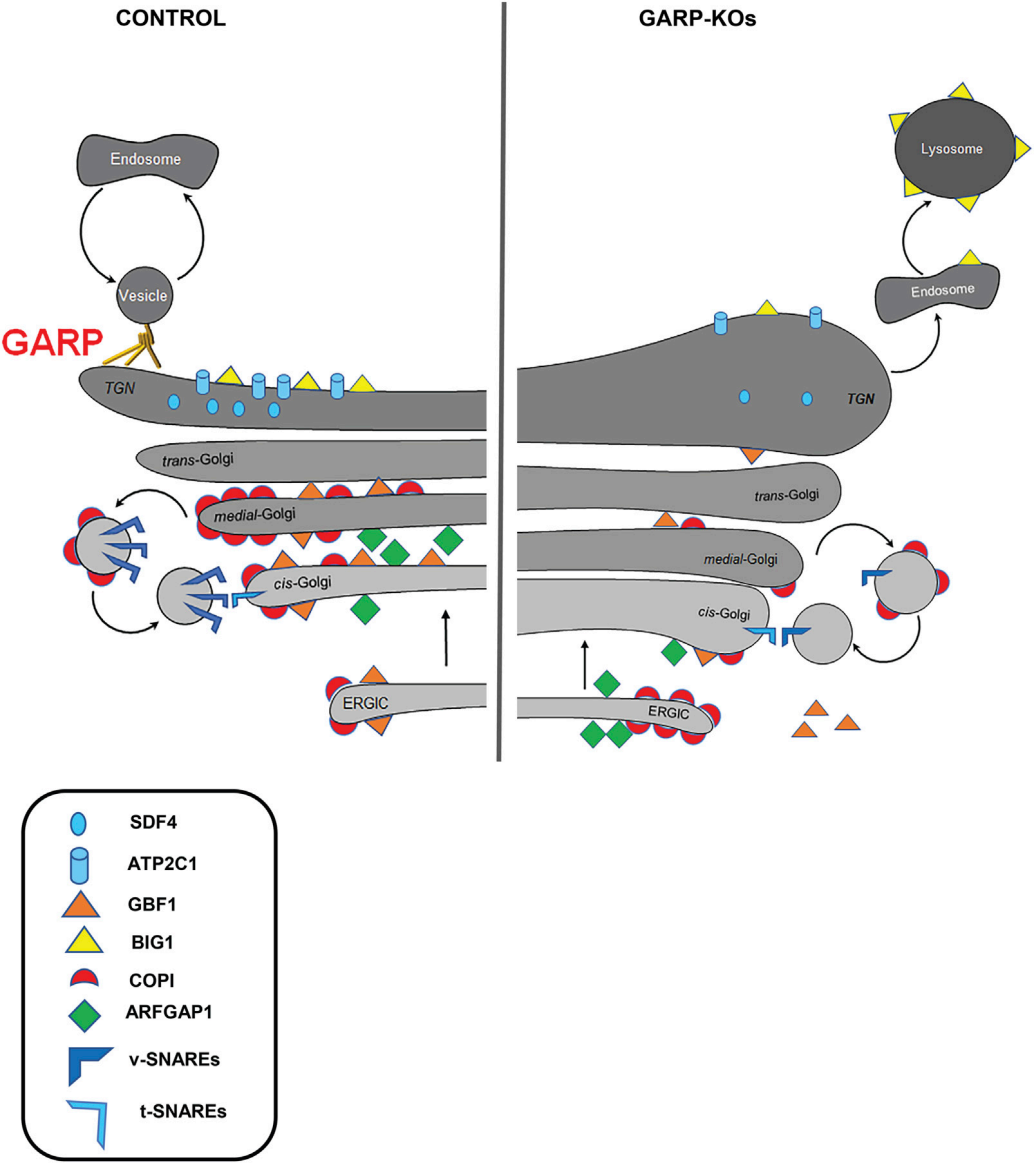
GARP dysfunction alters Golgi homeostasis and Golgi trafficking machineries. A cartoon depicting Golgi trafficking machinery in WT and GARP-KO cells. Proper maintenance of Golgi structure, calcium channel and Golgi trafficking machinery including SNAREs, COPI coats, ARFGEFs in control cells (Left panel). Disruption of the Golgi structure, calcium channel, and Golgi trafficking machineries in GARP-KO cells (right panel).

In this study, we have extended our investigation of the Golgi defects in human cells. The summary of observed GARP-related Golgi defects is presented in Figure 12. Microscopy and proteomic approaches revealed severe alterations of the Golgi structure in GARP-KO cells that coincided with a significant depletion of a subset of Golgi resident proteins. Mutant phenotypes were rescued by the expression of the deleted GARP subunit at endogenous level and most of these phenotypes were similar in both VPS53KO and VPS54KO cells. Golgi appeared more expanded in VPS54KO cells, while the abundance of Golgi resident proteins SDF4, ATP2C1, and GOSR1 was more severely decreased in VPS53KO cells. Subtle variations between VPS53 and VPS54 GARP mutants were likely because VPS53 is a common component of both the GARP and EARP tethering complexes, whereas VPS54 is a unique component of the GARP complex (Schindler et al., 2015).

Although the GARP is TGN-localized vesicle-tethering machinery, both *trans*- and *cis*-Golgi compartments are affected in GARP-KO cells. A subpopulation of GARP-KO cells also showed significant relocalization of ERGIC-53 from the Golgi area (A.K. unpublished observation), indicating that ERGIC was also affected by GARP depletion. Alterations in Golgi structure could be caused by the missorting and lysosomal degradation of recycling parts of GARP-dependent trafficking machinery, although some of these components could be degraded by the proteasome (Eisenberg-Lerner et al., 2020). Importantly, Golgi morphological alterations in GARP deficient cells (volume expansion of all subcompartments and lack of vesicles) were distinct from the severe fragmented Golgi phenotype observed in cells depleted for the other two Golgi vesicle tethering complexes COG (Zolov and Lupashin, 2005; Blackburn et al., 2018; Liu et al., 2019) and ZW10/Dsl10 (Sun et al., 2007; Liu et al., 2019), indicating that GARP depletion affects a specific set of Golgi proteins. Indeed, Golgi proteomics revealed a distinct set of Golgi resident proteins affected in GARP-KO cells. As predicted from our previous study (Khakurel et al., 2021), the set of GARP-sensitive proteins includes several glycosylation enzymes. We also discovered that KO of GARP complex subunits affects the calcium pump ATP2C1 as well as calcium binding protein SDF4. Furthermore, we found that localization of key elements of intra-Golgi trafficking machineries including v-SNAREs, COPI proteins, ARFGAP1 and ARFGEFs are also severely affected in GARP-KO cells.

SDF4/Cab45 is a calcium-binding luminal Golgi resident protein that is responsible for sorting specific cargo proteins at the TGN (Crevenna et al., 2016; Blank and von Blume, 2017; Deng et al., 2018; Hecht et al., 2020; Lebreton et al., 2021). SDF4 depletion in the Golgi can be due to several reasons. First, SDF4 could be cycling between TGN and the endosomal compartment in a GARP-dependent manner and fail to return to TGN in GARP-depleted cells. Second, SDF4 retention in the TGN requires a high Ca^2+^ concentration in the Golgi lumen (von Blume et al., 2012), which could be altered in GARP-KO cells. Interestingly, in both scenarios, we expected to find an increased secretion of SDF4 in GARP-KO cells. The third possibility is that GARP deficiency is forcing the displacement of SDF4 into other secretory/endolysosomal compartments. Our preliminary results (A.K. unpublished data) indicate a decrease of SDF4 in the secretome from GARP-KO cells which suggests that the decrease in SDF4 cellular levels is likely caused by its missorting and intracellular degradation. A decrease in SDF4 expression is likely to relate to the depletion of Golgi calcium pump ATP2C1/SPCA1. ATP2C1 pumps Ca^2+^ into the TGN lumen and defect in ATP2C1 results in missorting of secretory cargo (von Blume et al., 2011; Kienzle et al., 2014). Mutation of ATP2C1 gene is associated with Hailey-Hailey disease (Li et al., 2016; Miyazaki et al., 2022). Depletion of ATP2C1 in GARP-KOs could also be connected to the alteration in TGN morphology. In support of this hypothesis, Micaroni et al. showed that correct ATP2C1 functioning is critical for intra-Golgi trafficking and maintenance of Golgi structure (Sepúlveda et al., 2009; Lissandron et al., 2010; Micaroni et al., 2010).

GARP was shown to regulate formation and/or stability of TGN STX16/STX6/VTI1A/VAMP4 SNARE complex (Pérez-Victoria and Bonifacino, 2009; Emperador-Melero et al., 2019), but surprisingly we did not find any components of STX16 complex among proteins depleted from the Golgi membranes in GARP-KO cells. Instead, GOSR1, v-SNARE of intra-Golgi STX5/GOSR1/BET1L/YKT6 (Xu et al., 2002) complex was severely depleted in GARP-KO cells. BET1L, most likely because of its small size, was not detected in the proteomic studies, but our analysis indicated mislocalization of this Golgi v-SNARE in GARP-KO cells. The total BET1L protein level was also decreased in GARP-deficient cells (Khakurel et al., 2021). This indicates that GARP could be involved in regulation of STX5/GOSR1/BET1L/YKT6 SNARE complex. Interestingly, this SNARE complex has been implicated not only in intra-Golgi (Linders et al., 2019; D’Souza et al., 2021) but also in the endosome to TGN transport (Tai et al., 2004). Therefore, one explanation for the loss of GOSR1 and BET1L in GARP deficient cells is their inability to recycle back to the Golgi from the endosomal compartment. Importantly, the complete knock-out of GOSR1 or BET1L was less deleterious to cells as compared to the loss of the GARP complex, indicating that the loss of v-SNARE alone could not explain all Golgi phenotypes observed in GARP deficient cells.

Indeed, we discovered that the entire COPI vesicle budding machinery is significantly mislocalized in GARP-deficient cells to off-Golgi compartments and cytosol. The most prominent GARP-dependent displacement was observed for the COPI adaptor protein GOLPH3. GOLPH3 plays a crucial role in sorting a subset of Golgi resident glycosyltransferases back to Golgi by binding to the cytoplasmic tails of Golgi glycosyltransferases to package them into recycling COPI vesicles (Schmitz et al., 2008; Tu et al., 2008; Frappaolo et al., 2020; Lowe, 2021; Sardana et al., 2021; Welch et al., 2021), and its displacement from the Golgi in GARP deficient cells could be responsible for some glycosylation defects in mutant cells. Displacement of GOLPH3, however, could not explain the depletion of B4GALT1 and MGAT1 in GARP-KO cells (Khakurel et al., 2021), since GOLPH3 is not responsible for their Golgi retention. So, our next question was what would be the reason for the decrease in Golgi localization of COPI coats?

GBF1 is a *cis*-Golgi ARFGEF that activates ARF GTPases and takes part in COPI recruitment, Golgi integrity and secretory traffic whereas inactivation or depletion of GBF1 inhibits these processes (García-Mata et al., 2003; Szul et al., 2007; Kaczmarek et al., 2017). We were unable to investigate the changes in localization of the endogenous Arf1 in GARP-KO cells, but significant displacement of GBF1 is likely to cause Arf1 and COPI depletion from the Golgi membranes.

Not only GBF1 was depleted from the Golgi in GARP-KOs, but the localization of BIG1, an ARFGEF known to function at *trans*-Golgi and endosomes (Boal and Stephens, 2010) was also altered, resulting in BIG1 relocation to endolysosomal compartments. BIG1 mislocalization is consistent with the finding that inactivation of GBF1 by inserting mutation or treating with GBF1-selective drug golgicide (GCA) inhibited Golgi membrane recruitment of BIG1 and BIG2 (Lowery et al., 2013).

One of the reasons for the depletion of COPI machinery in the Golgi could be an alteration of membrane lipid content in GARP-KO cells. It has been demonstrated previously that inhibitors of PI4P synthesis prevent the recruitment of GBF1 to Golgi membranes (Dumaresq-Doiron et al., 2010). PI4P is also essential for GOLPH3 binding to membranes (Rahajeng et al., 2019). Indeed, MS analysis revealed a significant decrease in Golgi-associated PI4K2a kinase, supporting the possibility that PI4P Golgi content is altered in GARP-KO cells. In addition, GARP mutations in yeast and mouse models result in sphingolipid abnormalities (Fröhlich et al., 2015; Petit et al., 2020). The effect of GARP depletion on the lipid content of Golgi in human cells will be investigated in the future.

Although the exact mechanisms of GARP-dependent relocalization of COPI and COPI-associated proteins will require additional investigation, we propose that the dysregulation of COPI machinery along with depletion of intra-Golgi v-SNAREs and Ca^2+^ homeostasis are the major driving factors for the alterations of Golgi structure, decreased expression of resident proteins and glycosylation defects in GARP deficient cells.

## Data Availability

The datasets presented in this study can be found in online repositories. The names of the repository/repositories and accession number(s) can be found in the article/Supplementary Material.
