# Supplementary material for: GARP dysfunction results in COPI displacement, depletion of Golgi v-SNAREs and calcium homeostasis proteins
Source: Front Cell Dev Biol. 2022 Dec 12;10:1066504. doi: 10.3389/fcell.2022.1066504 (PMC9791199; doi:10.3389/fcell.2022.1066504)

Supplementary information files

GARP dysfunction results in COPI displacement, depletion of Golgi v-SNAREs and calcium homeostasis proteins

Amrita Khakurel, Tetyana Kudlyk, Irina Pokrovskaya, Zinia D'Souza and Vladimir V. Lupashin*

Department of Physiology and Cell Biology, University of Arkansas for Medical Sciences, Little Rock, AR, United States

Supplemental figure 1. Uncropped Western blot images of the representative blots used in the manuscript.

Supplemental Table 1: The excel file included in supplemental materials is the proteomics data. The proteomics data is available in PRIDE database with accession number PXD035659.

Uncropped images of Western Blot

Figure 2B

B4GALT1

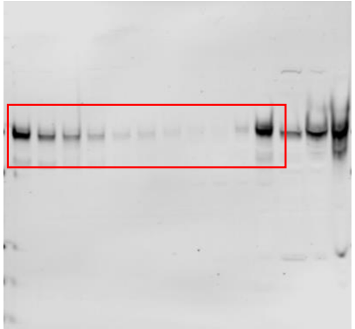

Figure 3D

SDF4

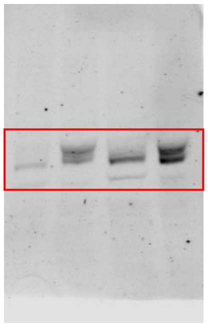

β- actin

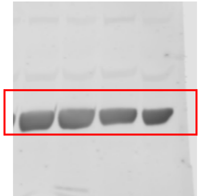

Figure 3F

ATP2C1

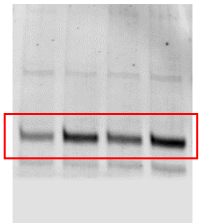

β- actin

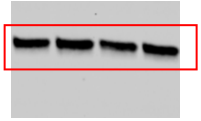

Figure 4C

GOSR1

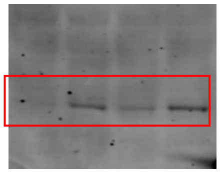

β- actin

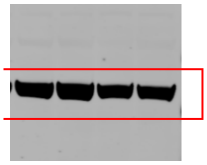

Figure 5A

BET1L

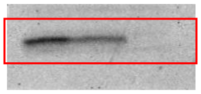

β- actin

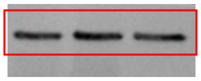

Figure 5A

GOSR1

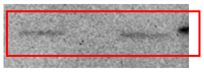

Figure 5B

SDF4

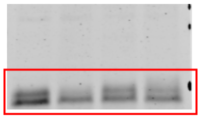

β- actin

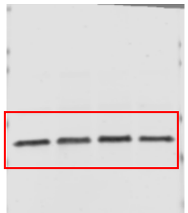

Figure 5D

B4GALT1

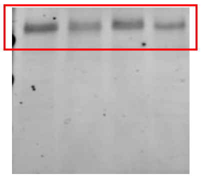

β- actin

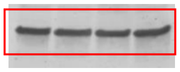

Figure 5F

TGN46

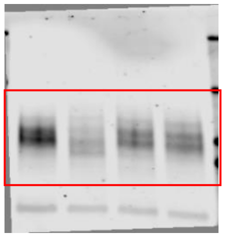

β- actin

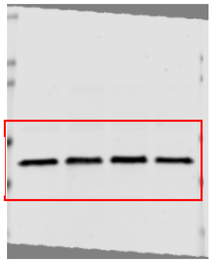

Figure 7E

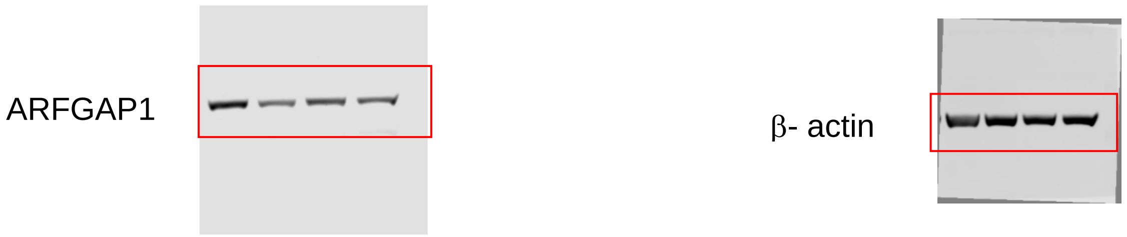

Figure 9C

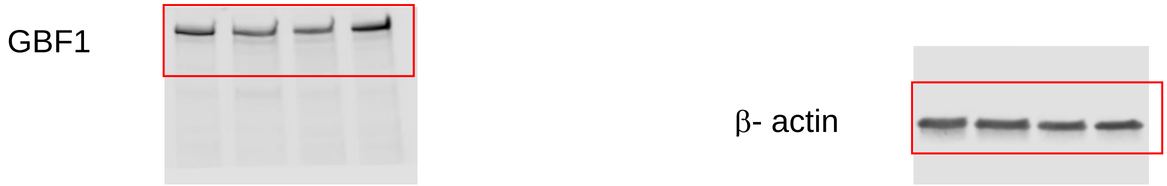

Supplement: Supplementary file 1 [file Presentation1.pdf]
